# Anti-Tumorigenic Effect of a Novel Derivative of 2-Hydroxyoleic Acid and the Endocannabinoid Anandamide on Neuroblastoma Cells

**DOI:** 10.3390/biomedicines10071552

**Published:** 2022-06-29

**Authors:** Hana Golan, Raphael Mechoulam, Reem Smoum, Efrat Cohen-Zada, Sara Pri-Chen, Sapir Wiener, Igor Grinberg, Dekel D. Bar-Lev, Christeeneh G. Haj, Tamar Fisher, Amos Toren

**Affiliations:** 1 Pediatric Hematology Oncology Research Laboratory, Cancer Research Center, The Chaim Sheba Medical Center, Tel-Hashomer, Ramat Gan 52621, Israel; hana.golan@sheba.health.gov.il (H.G.); efratco1@gmail.com (E.C.-Z.); sara.prichen@sheba.health.gov.il (S.P.-C.); sapirneeman24@gmail.com (S.W.); igor.grinberg@sheba.health.gov.il (I.G.); dekeldov.barlev@sheba.health.gov.il (D.D.B.-L.); tamar.fisher@sheba.health.gov.il (T.F.); 2 Department of Pediatric Hematology Oncology, The Edmond and Lily Safra Children’s Hospital, The Chaim Sheba Medical Center, Tel-Hashomer, Ramat Gan 52621, Israel; 3 Sackler Faculty of Medicine, Tel-Aviv University, Tel-Aviv 6997801, Israel; 4 Medicinal Chemistry Laboratory, The Institute for Drug Research, School of Pharmacy, Faculty of Medicine, The Hebrew University of Jerusalem, Jerusalem 9112001, Israel; raphaelm@ekmd.huji.ac.il (R.M.); reems@ekmd.huji.ac.il (R.S.); christeeneh@ekmd.huji.ac.il (C.G.H.)

**Keywords:** 2-hydroxy oleic acid, anandamide, anti Bcl2, endocannabinoid system, membrane lipid therapy, neuroblastoma

## Abstract

Modulation of the endogenous cannabinoid system has been suggested as a potential anticancer strategy. In the search for novel and less toxic therapeutic options, structural modifications of the endocannabinoid anandamide and the synthetic derivative of oleic acid, Minerval (HU-600), were done to obtain 2-hydroxy oleic acid ethanolamide (HU-585), which is an HU-600 derivative with the anandamide side chain. We showed that treatment of SK-N-SH neuroblastoma cells with HU-585 induced a better anti-tumorigenic effect in comparison to HU-600 as evidenced by 3-[4,5-dimethylthiazole-2-yl]-2,5-diphenyltetrazolium bromide assay, colony-forming assay, and migration assay. Moreover, HU-585 demonstrated pro-apoptotic properties shown by increased levels of activated caspase-3 following treatment and a better senescence induction effect in comparison to HU-600, as demonstrated by increased activity of lysosomal β-galactosidase. Finally, we observed that combined treatment of HU-585 with the senolytic drugs ABT-263 in vitro, and ABT-737 in vivo resulted in enhanced anti-proliferative effects and reduced neuroblastoma xenograft growth in comparison to treatment with HU-585 alone. Based on these results, we suggest that HU-585 is a pro-apoptotic and senescence-inducing compound, better than HU-600. Hence, it may be a beneficial option for the treatment of resistant neuroblastoma especially when combined with senolytic drugs that enhance its anti-tumorigenic effects.

## 1. Introduction

Neuroblastoma (NBL) is the most common extracranial solid tumor of childhood. Children with high-risk neuroblastoma currently have long-term survival rates under 50% despite intensive, multimodal treatment regimens that include chemotherapy, surgical tumor resection, autologous stem cell transplantation, radiation therapy, and maintenance immunotherapy combined with 13-*cis*-retinoic acid [1,2]. In addition, the aggressive chemo-radiotherapy used for the treatment of these children is associated with severe side effects and multi-organ damage [3]. Therefore, new, less toxic therapeutic combinations directed at relevant targets are needed for these children to reduce relapse rates and improve survival. 

The cannabinoids are a group of more than 100 chemically related compounds found in the marijuana plant *Cannabis sativa*, that have been found to possess diverse pharmacological activities in cancer, including cytostatic, apoptotic, and antiangiogenic effects [4]. Tetrahydrocannabinol (THC), the main psychoactive constituent in *Cannabis sativa*, acts mainly through the activation of specific cannabinoid receptors CB1 and CB2 and thus mimics the binding of the animal endogenous cannabinoids (named endocannabinoids) [5,6,7]. Endocannabinoids are known to participate in many biological processes in the immune, respiratory, circulatory, and reproductive systems. On a cellular level, they have been shown to modulate cell proliferation, viability, and differentiation [8]. The cytotoxicity of endocannabinoids on tumoral cells has been frequently reported, hence, their potential use in the treatment of malignant diseases [9]. Several different mechanisms have been implicated in the anti-tumorigenic actions of endocannabinoids and include cytotoxic or cytostatic effects, apoptosis induction, and anti-metastatic effects, such as inhibition of neo-angiogenesis and tumor cell migration [10]. One of the best-known endocannabinoids with anti-tumorigenic effects is anandamide (AEA). AEA has been shown to inhibit cholangiocarcinoma growth [11], to exert cytotoxic and antiproliferative effects on colorectal carcinoma cells [12], and to cause apoptosis of osteosarcoma cells [13] and glioma cells [14]. 

AEA is the ethanolamide of the fatty acid arachidonic acid. The anti-cancer drug Minerval is a 2-hydroxy derivative of another fatty acid, oleic acid. Minerval (HU-600) is one of the most studied synthetic lipid compounds that was shown to be safe and effective in patients with glioma and other advanced solid tumors [15]. In contrast to most anticancer drugs, HU-600 targets the plasma membrane and mediates its anti-tumor effect by affecting the biophysical properties of membranes [16,17,18].

Based on the anti-cancer effects of HU-600 and AEA, we assumed that a molecule that is a derivative of both compounds may be a novel anti-cancer drug with a promising anticancer therapeutic profile. Hence, structural modification of HU-600 was done to obtain 2-hydroxy-oleic acid (2-OHOA) ethanolamide (HU-585), a novel compound, which is an HU-600 derivative with the AEA side chain.

To further our research on this subject [19], we explored and compared the anti-tumorigenic effects of HU-600 and its novel derivate HU-585 on the NBL cell line. The results obtained in our study indicate that of the two compounds tested, HU-585 was indeed more effective on the NBL cell line in comparison to HU-600. Furthermore, HU-585 demonstrated pro-apoptotic and senescence-inducing properties, and combined treatment with senolytic drugs further enhanced its anti-tumorigenic effect. Our findings add contemporary information, attractive strategy, and an effective and less toxic therapeutic option for the treatment of refractory NBL.

## 2. Materials and Methods

### 2.1. Preparation of 2-Hydroxyoleoyl Ethanolamide (HU-585)

The 2-hydroxy oleic acid (HU-600) was synthesized according to Lazarus et al. [20]. The ethanolamide derivative (HU-585) of HU-600 was prepared according to the following procedure: To a solution of 2-hydroxyoleic acid sodium salt (100 mg, 0.3125 mmol) and N, N-dimethylformamide (23.65 µL, 0.3125 mmol) in dry methylene chloride (4.7 mL) was added dropwise oxalyl chloride (2.0 M solution in methylene chloride, 0.312 mL, 0.62 mmol) under nitrogen atmosphere at 0–5 °C. The reaction mixture was stirred for 10 min, and then the solvent was evaporated under nitrogen flow. The crude material in methylene chloride (4.7 mL) was added to an ice-cold solution of ethanolamide (0.214 mL, 3.55 mmol) in methylene chloride (4.7 mL). The reaction mixture was stirred for 10 min, then it was washed with water (3 × 20 mL) and dried (MgSO_4_), and the solvent was evaporated under reduced pressure. The residue was chromatographed on silica gel. White solid, melting point: 63 °C, NMR (CDCl3, ppm): 5.37 [m, 2H]; 4.15 [dd, 1H]; 3.75 [m, 2H]; 3.52–3.45 [m, 2H]; 2.04 [m, 4H]; 1.85–1.66 [m, 2H]; 1.44–1.29 [m, 20H]; 0.92 [t, 3H]. LC-MS (+p) = 342.2.

### 2.2. Cell Culture

Human neuroblastoma cell line SK-N-SH was obtained from ATCC (Manassas, VA, USA). SK-N-SH cells were cultured in Eagle minimum essential medium (ATCC), supplemented with 10% fetal bovine serum (FBS) and 100 U/mL penicillin—streptomycin (Gibco, Paisley, UK). Fibroblasts were obtained from dermal human fibroblasts, per protocol #7044 (approved by the Institutional Review Board at Sheba Medical Center, Tel Hashomer, Ramat Gan, Israel). Fibroblasts cells were cultured in high glucose DMEM supplemented with 20% FBS, penicillin-streptomycin, 1% non-essential amino acids and 0.2% β-mercaptoethanol (Invitrogen, Life Technologies, Grand Island, NY, USA). Normal human astrocytes (HA) obtained from Science Cell Research Laboratories (Carlsbad, CA, USA). HA cells were cultured in HA medium (Catalog #1801) with supplement from Science Cell Research Laboratories (Carlsbad, CA, USA). All cells were routinely tested for the presence of mycoplasma. The cell lines were cultured at 37 °C in a humidified atmosphere containing 5% CO_2_. As they approached confluence, the cells were sub-cultured following treatment with 6% trypsin-EDTA.

### 2.3. MTT Test

The effect of HU-600 and HU-585 on the SK-N-SH NBL cell line was studied using the 3-(4, 5-dimethylthiazol-2-yl)-2,5diphenyltetrazolium bromide (MTT) assay (Sigma-Aldrich Co., St. Louis, MO, USA). SK-N-SH cells (4 × 10^3^ cells/well) were plated (200 µL) in triplicates in flat bottom 96-well plates in appropriate medium as mentioned above. The cells were allowed to adhere to the plate surface overnight and then cultured with increasing doses of HU-600 or HU-585 (0–200 µM) for 72 h. Cell viability was then determined using MTT assay, which measures reduction of MTT to formazan by mitochondria of viable cells. Formazan was measured spectrophotometrically by absorption at 560 nm in a PowerWaveX^TM^ (BioTek, Santa Clara, CA, USA) plate reader. All experiments were repeated at least 3 times. Cell morphologies were assessed daily by light microscopy.

### 2.4. Colony-Forming Assay (CFA)

CFA was used to determine the effect of HU-600 and HU-585 treatment on the ability of a single cell to grow into a colony. SK-N-SH cells (500 cells/well) were plated in triplicates in 6-well plates (3 mL) and were treated according to treatment regimen mentioned below with 12.5, 37.5, and 75 µM of HU-600 or HU-585 for 14 days. Subsequently, colonies were fixed with ethanol 70%, stained with crystal violet solution (0.5% *w*/*v*), rinsed extensively by tap water, dried, and counted using EPSON scan software. Colonies were counted using ImageJ software.

### 2.5. Cell Migration Assay

Wound healing assay was performed to compare the effect of HU-600 and HU-585 on cell migration ability. SK-N-SH cells (1 × 10^6^ cells/well) were plated in duplicates in 6-well plates (3 mL) and were allowed to adhere overnight, then treated with 75 µM of HU-600 or HU-585, and were allowed to grow into a confluent cell monolayer. Cells then were starved using starvation medium (FBS free) overnight. A single scratch along each well was made by micropipette, fresh medium was supplemented, and cells were incubated for 24 h. Cell migration was quantified by taking pictures with a regular inverted microscope at two time points: right after scratching and 24 h later. Gap intervals were measured using ImageJ software, and the percentage of migration and gap closure was calculated using the formula:

$$\%\;migration=\frac{average\;wound\;width\;0h-average\;wound\;width\;24h}{\;average\;wound\;width\;0h}\times 100$$

### 2.6. Western Blot Assay

To assess apoptotic activity following treatment with HU-585, caspase-3 and Bcl-2 protein levels were evaluated by western blot assay. One day before HU-585 treatment (100 µM for 24 h and 48 h), cells were plated (1 × 10^6^ cells per 9-cm plate). Cells were harvested and proteins were extracted with RIPA buffer supplemented with phosphatase and protease inhibitors (Sigma–Aldrich). Protein concentrations were calibrated using the BCA Protein Assay Reagent Kit (Pierce, Rockford, IL, USA). Equal amounts of protein (30 μg) were loaded onto 12.5% SDS-PAGE gels and transferred onto nitrocellulose membranes (Bio-Rad, Rishon Le Zion, Israel). The blots were reacted using caspase-3 (9662S) or Bcl-2 (D55G8, 4223S) rabbit monoclonal antibody (Cell Signaling Technology, Danvers, MA, USA) as the primary antibody. The secondary antibody, horseradish peroxidase conjugated goat anti-rabbit antibody (Jackson ImmunoResearch Laboratories, Farmington, CT, USA), was detected by chemiluminescence. Signals were detected using an ECL Kit (CYANAGEN, Bologna, Italy) and visualized using the ChemiDocTM MP Imaging System. Quantification of caspase-3 and Bcl-2 was done by Image Lab software.

### 2.7. Senescence-Associated β-Galactosidase Activity Assay

Senescence-associated β-galactosidase (SA-β-gal) activity was measured with a β-galactosidase staining kit (Senescence B-Galactosidase Staining KIT, Cell Signaling Technology, #9860) according to the manufacturer’s instructions. Briefly, SK-N-SH cells (5 × 10^4^ cells\well) were plated in 6-well plates (3 mL) and treated according to the previously described treatment regimen [19] with 50, 75 and 100 µM of HU-600 or HU-585 for 48 h and then fixed and incubated overnight at 37 °C in CO_2_ free environment. Accumulation of a distinctive blue color in senescent cells was then observed by microscope (Olympus Scientific Solutions, Waltham, MA, USA). Pictures of three representative fields of each well were taken, blue colored cells were counted by ImageJ software, and the percentage of β-galactosidase positive cells was determined and normalizing to the control.

### 2.8. In Vitro Senolytic Studies

In vitro senolytic studies were performed using Navitoclax (ABT-263) dissolved in DMSO (Sigma-Aldrich, Rehovot, Israel). SK-N-SH cells (2 × 10^4^ cells/well) were plated in triplicates in 96-well plates (200 µL) and were cultured according to the previously described regimen, with increasing concentrations of HU-600 or HU-585 (0–200 µM) for 48 h. Subsequently, cells were cultured with ABT-263 (2.5 µM) for 24 h, then an MTT test was performed according to the MTT assay mentioned above. ABT-263 concentration was chosen following several dose response MTT assays. The maximal dose with no effect on survival was 2.5 µM (data not shown).

### 2.9. Murine Xenograft Therapeutic Studies

In vivo experiments were carried out according to protocols approved by the Ethical Committee of the Chaim Sheba Medical Center. Female athymic nude mice (Foxn1nu) 6–8 weeks of age (ENVIGO RMS, Jerusalem, Israel) were used for the tumor xenograft (Xn) model. In total, 5 × 10^6^ SK-N-SH mCherry expressing cells were subcutaneously inoculated in the right flank of each mouse. Following cell injection, tumor burden was determined once a week by a Spectrum Animals in vivo imaging system (IVIS®), and the mice were allocated into four homogeneous groups according to the intensity of the average total radiant efficiency signal measured by IVIS: Control vehicle; HU-585 (120 mg/kg); ABT-737 (75 mg/kg); combined treatment with HU-585 (120 mg/kg) and ABT-737 (75 mg/kg), (*n* = 8 per group). ABT-737 (S1002, Selleckchem, Houston, TX, USA) was formulated in a mixture of 30% propylene glycol, 5% Tween 80, and 65% DsW (5% dextrose in DDW). Treatment started 4 weeks following cell injection and was administered intraperitoneally (IP) once daily for a total of 21 days. Tumor burden was determined once a week by the IVIS system and by external electronic caliper measurements throughout treatments. Tumor volumes were calculated by the following formula: A × B^2^/2, where A is the greatest diameter, and B is the diameter perpendicular to A. At the end of treatment, the animals were then euthanized, and tumor Xns were immediately removed, weighed, stored, and fixed.

### 2.10. Statistical Analysis

An estimating equations (GEE) test was used to evaluate significant differences in all in vitro experiments except for apoptosis assay in which Two-way ANOVA was used. The Kruskal-Wallis test was performed with the post hoc Dunn’s multiple comparisons to evaluate significant differences in the growth rate of xenografts between treatment groups. All analyses were performed using the IBM SPSS Statistics software application (version 24: IBM, Armonk, NY, USA). Excluding MTT analysis, all results are shown as means ± SE. *p* < 0.05 was considered to indicate statistical significance.

## 3. Results

### 3.1. Structural Modification of HU-600

**Figure 1:**
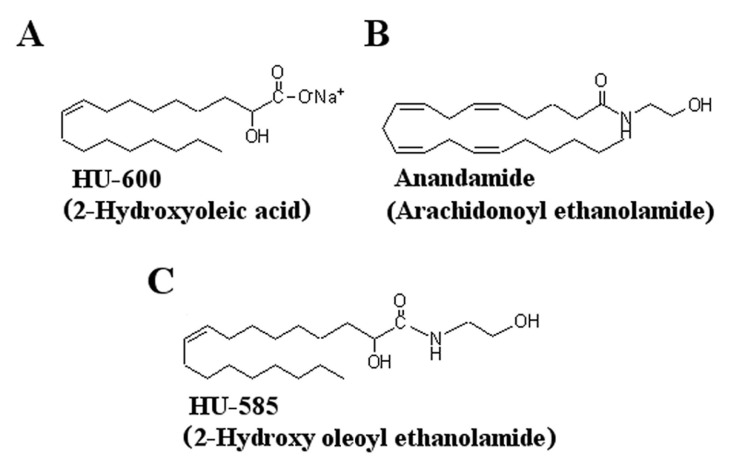
Structures of the synthetic compounds tested for anticancer activity. Structures of (**A**) HU−600 (Minerval, 2−hydroxyoleic acid), (**B**) anandamide (AEA) and (**C**) HU−585 that was obtained by structural modification and the addition of AEA side chain to HU−600.

The syntheses 2-OHOA ethanolamide (HU-585) was done by the addition of anandamide side chain to 2-OHOA (HU-600) (Figure 1).

### 3.2. HU-585 Induces Cell Growth Inhibition, Reduced Colony Formation, and Reduced Migration in the Neuroblastoma Cell Line SK-N-SH In Vitro

**Figure 2:**
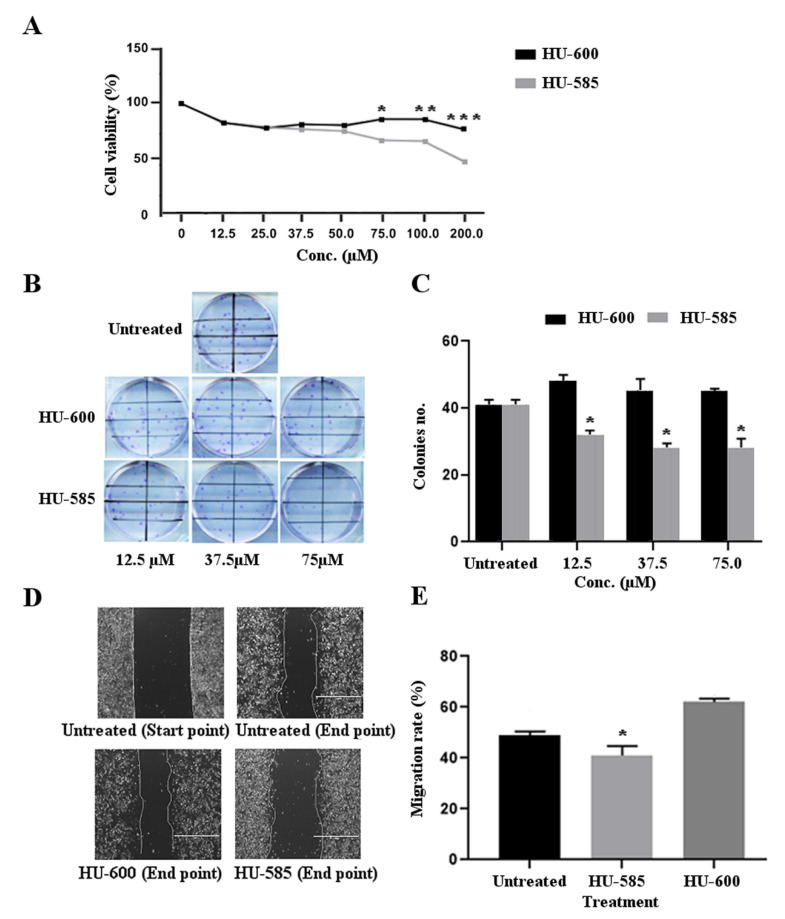
Anti-tumorigenic effects of HU-585 and HU-600. (**A**) Cell viability, measured with MTT assay in SK-N-SH cell line following treatment with HU-585 and HU-600. The cells were plated in 96-well plates and treated with increasing concentrations of HU-600 or HU-585 for 72 h. A significant decrease in cell viability was detected for both compounds at concentration of 12.5 µM (16.7%, *p* < 0.05). A better effect in cell viability reduction was obtained for HU-585 at concentration 75 µM and above. The cell viability reduction for HU-585 and HU-600 at 75 µM was 33.5% and 14.4%, respectively (* *p* = 0.03), at 100 µM, 34.3% and 14.7%, respectively (** *p* = 0.02), and at 200 µM, 52.2% and 23.2%, respectively (*** *p* = 0.01). Data are expressed as percentage of the vehicle control and are the mean of pooled results from several experiments (*n* = 6) performed in triplicate. Statistical significance was determined by GEE test. * *p* < 0.05 compared to HU-585 (1.15 < SE values < 1.99). (**B**,**C**) The effect of HU-585 and HU-600 on the colony formation ability of SK-N-SH cells showing a better effect of HU-585. The cells were plated in 6-well plates and treated with 12.5 μM, 37.5 μM and 75 μM of HU-585 or HU-600. Cells were fixed and stained with crystal violet 14 days later. Colonies were counted using ImageJ software. (**B**) Scanned image of representative wells showing different levels of colony formation in SK-N-SH treated cells. (**C**) Representation of the quantified number of colonies in increasing concentrations of the treatment used. Statistical significance was determined by GEE test, * *p* < 0.05 (*n* = 3, performed in duplicates). (**D**,**E**) Migration rate of SK-N-SH cells was decreased following treatment with HU-585. Using ImageJ software, pre-migration (0 h) and post-migration (24 h) images of untreated and following treatment with 75 μM of HU-585 or 75 μM of HU-600 were taken (**D**). Treatment with HU-585 resulted in a better inhibitory effect of the migration rate in comparison to untreated and to HU-600 treated cells. Migration rate of the cells was quantified as the average percentage of gap closure following treatment (**E**). Data is reported as mean ± SE of triplicates. Statistical significance was determined by GEE test, * *p* ≤ 0.05 compared to the two other group (*n* = 3, performed in duplicates).

In order to evaluate the antitumorigenic effects of HU-600 and its derivative, HU-585, on SK-N-SH cells, we used MTT, CFU, and migration assays (Figure 2). The choice of concentrations used in these assays (12.5–200 µM) is based on previous data showing that the IC_50_ of HU600 for most cancer cells studied is in the range of 30–250 µM [16,21,22]. A similar and significant dose-dependent decrease in cell viability was demonstrated by both compounds at a concentration of 12.5 µM (16.7%, *p* < 0.05). The effect on cell viability reduction was significantly better for HU-585 treatment in comparison to HU-600 treatment at concentration of 75 µM and above: 33.5% vs. 14.4% at 75 µM; 34.3% vs. 14.7% at 100 µM; 52.2% vs. 23.2% at 200 µM, respectively. * *p* = 0.03, ** *p* = 0.02, *** *p* = 0.01, respectively (Figure 2A).

Cologenic assay was used to determine the effect of HU-600 and HU-585 treatment on the cellular cologenic potential of SK-N-SH cells (Figure 2B,C). The results show a significant reduction in the number of colonies formed following HU-585 treatment in comparison to untreated cells (mean 29.2 colonies/field vs. 40.55 colonies/field respectively, *p* < 0.001). The significant difference between HU-585 treatment and untreated cells was observed in all concentrations used (12.5 µM, 37.5 µM & 75.5 µM, *p* < 0.05) (Figure 2C). A similar effect of reduction in colony formation was not obtained following treatment with HU-600.

Finally, to assess the effect of HU-600 and HU-585 treatment on SK-N-SH cell migration ability, a wound healing assay was performed. The results show a significant reduction in migration rate of cells treated with 75.5 µM of HU-585 in comparison to untreated cells. Decrease in migration rate following treatment with HU-600 was not observed (Figure 2D,E).

Taken together, these results provide evidence that both HU-585 and HU-600 have antitumorigenic effects as demonstrated by reduced cells viability following treatment. However, the new compound HU-585 obtained by structural modification of HU-600, has a better anti-tumorigenic effect in the MTT assay at concentration of 75 μM and above and in all the additional in vitro studies that were performed.

### 3.3. Apoptotic Cell Death and Senescence Following HU-585 Treatment in SK-N-SH Cells

To verify our hypothesis that HU-585 induced reduction in NB cell viability was indeed due to apoptotic cell death, we first examined the morphological changes following HU-585 treatment. Microscopic analysis showed that treatment with 75 µM and 100 µM of HU-585 affected cell morphology and increased the number of cells that had lost their normal shape and became rounded, swollen and floated in the medium (data not shown). These results confirmed that HU-585 treatment might induce the appearance of typical features of apoptosis. Next, we used caspase assay and Bcl-2 levels following treatment to better evaluate the apoptotic activity of HU-585. Staurosporine-treated cells were used as the positive control to apoptosis.

**Figure 3:**
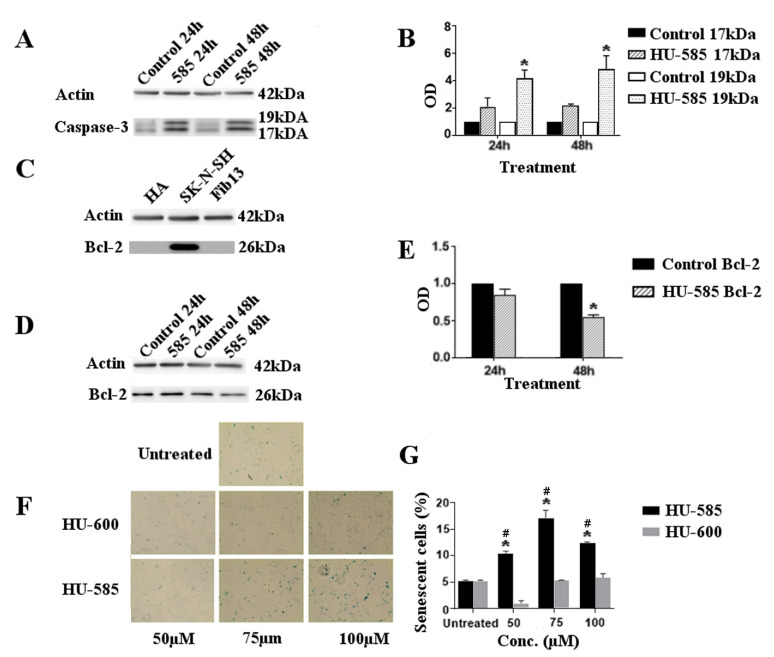
Growth suppression by HU-585 is mediated by apoptosis and senescence. (**A**–**E**) Apoptotic effects of HU-585. (**A**) Caspase-3 assay. Western blot of caspase-3 (35 kDa) cleavage to 19 kDa and 17 kDa represents the apoptotic effect of HU-585. SK-N-SH cells were treated with 100 µM of HU-585 for 24 h and 48 h. (**B**) Quantification of cleaved caspase-3. Quantification was done by Image Lab software and representation for 24 h and 48 h of incubation are shown. Cleaved 19 kDa level increased significantly in a time-dependent manner in comparison to its control following treatment with HU-585. Statistical differences at 24 h and 48 h were determined by two-way ANOVA (* *p* < 0.01). (**C**) Bcl-2 protein levels. Western blot of Bcl-2 protein levels determination for human astrocyte cell line (HA), SK-N-SH cells and fibroblast cell line (as a “normal control”). High levels of Bcl-2 protein were detected in SK-N-SH cells compared to the other cell lines. (**D**,**E**) Apoptotic effects of HU-585 analyzed by Bcl-2 levels following treatment. (**D**) Western blot of Bcl-2 protein levels in SK-N-SH cells following treatment with 100 µM HU-585 for 24 h and 48 h. (**E**) Quantification of Bcl-2 protein. Quantification was done by Image Lab software following treatment with 100 µM of HU-585 for 24 h and 48 h. Results revealed that the level of Bcl-2 protein decreased in a time-dependent manner, reaching maximal and significant effect at 48 h. Statistical significance was determined by Unpaired *t*-Test (* *p* < 0.001). (**F**,**G**) Senescence effect of HU-585 analyzed by β-galactosidase staining. (**F**) Activity of β-galactosidase in SK-N-SH cells following HU-600 and HU-585 treatment was measured by β-galactosidase staining. SK-N-SH cells were plated in 6-well plates and treated with 50 μM, 75 μM and 100 μM of HU-600, HU-585 or no treatment as control. Positivity for β-galactosidase following 48 h of treatment represents the cells that are in senescence. (**G**) Representation of the quantified percentages of senescent cells. Results show an increased number of senescent cells following HU-585 treatment compared to HU-600 treatment and untreated cells. Data is reported as mean ± SE of triplicates. Statistical significance was determined by GEE test. * *p* < 0.05 compared to HU-600 and ^#^ *p* < 0.05 compared to untreated cells.

Treatment of SK-N-SH cells with 100 µM of HU-585 induced apoptosis as demonstrated by cleavage of caspase-3 represented by the appearance of activated 17 kDa and 19 kDa fragments on western blot (Figure 3A). The apoptotic effect was observed at 24 h and peaked at 48 h (Figure 3B). In order to evaluate Bcl-2 levels following treatment, we first determined its baseline levels in SK-N-SH cells in comparison to fibroblasts and human astrocyte cell line. Western blot revealed a high baseline level of Bcl-2 protein in the SK-N-SH cells in comparison to normal controls (Figure 3C). Treatment with 100 µM of HU-585 resulted in a decreased level of Bcl-2 with a better and significant effect at 48 h in comparison to 24 h (Figure 3D,E).

As senescence is generally regarded as a tumor suppressive process that evolves alongside apoptosis to suppress tumorigenesis, we next assessed the effect of treatment with HU-600 and HU-585 on cell senescence. β-Galactosidase activity was measured by blue-colored cell counting (Figure 3F). Results show that cellular senescence occurred at 48 h following treatment with HU-585, but not with HU-600 (Figure 3G).

Overall, these results provide evidence that treatment with HU-585 induces apoptosis and cellular senescence in SK-N-SH cells, while no such effect is demonstrated for HU-600 treatment.

### 3.4. Combined Treatment of HU-585 with Anti Bcl-2 Compounds ABT-263 or ABT-737 Results in Cell Growth Inhibition In Vitro and in Tumor Growth Delay In Vivo

**Figure 4:**
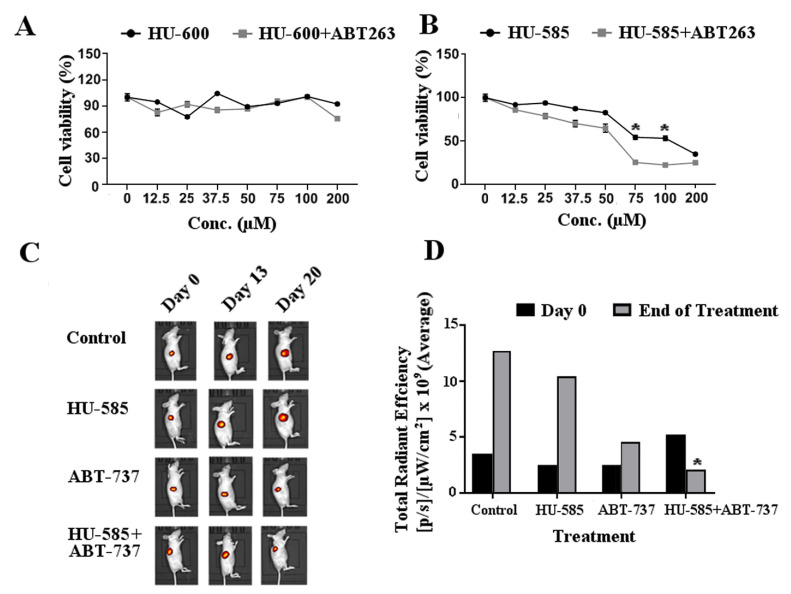
The combination of HU-585 with anti Bcl-2 compounds results in enhanced tumor growth delay in vitro and in vivo. (**A**,**B**) The effect of combination therapy of ABT-263 with HU-600 or HU-585 on SK-N-SH cells viability. The cells were plated in 96-well plate and treated with increasing concentrations of HU-600 (**A**) or HU-585 (**B**) for 72 h, with or without 2.5 µM of ABT-263. Viability measurements are shown by MTT assay and demonstrate a better effect of ABT-263 treatment combined with HU-585 in comparison to HU-585 alone. A similar effect was not obtained following combination of ABT-263 with HU-600. All values are normalized to control. Data are expressed as percentage of the vehicle control and are the mean of pooled results from several experiments performed in triplicate. Statistical significance was determined by GEE test, * *p* < 0.05 compared to HU-585 + ABT263 (0.86 < SE values < 4.22). (**C**,**D**) Combined treatment of ABT-737 with HU-585 inhibited tumor growth in mice model. A total of 5 × 10^6^ SK-N-SH expressing mCherry cells were subcutaneously inoculated in the right flank of nude mice. The mice were divided into four groups and given vehicle, HU-585 (120 mg/kg), ABT-737 (75 mg/kg) or combination of HU-585 with ABT-737 (120 mg/kg, 75 mg/kg, respectively) once daily by IP injections for 21 days. (**C**) Tumor burden was followed by IVIS system once a week during treatment. (**D**) Average total radiant efficiency signal was significantly lower following combined treatment with HU-585 and ABT-737 compared to control or HU585 alone. The Kruskal-Wallis test and the post hoc Dunn’s multiple comparisons test were used to evaluate significant differences in the growth rate of xenografts between treatment groups, * *p* < 0.05 compared to control and HU-585.

As we have shown that SK-N-SH cells express high levels of Bcl-2 (Figure 3C), which have been shown to be important for neuroblastoma survival, we wished to study the effect of combined treatment of HU-585 with anti Bcl-2 compounds. First, MTT assay was performed to evaluate the in vitro effect of combined treatment of ABT-263 with HU-585 or HU-600. The reduction in SK-N-SH cell viability treated with the combination of HU-600 with 2.5 µM of ABT-263 was not significantly different in comparison to HU-600 alone (Figure 4A). In contrast, treatment of the cells with combination of HU-585 and 2.5 µM of ABT-263 resulted in a significantly reduced viability when compared to treatment with HU-585 alone. This effect was dose-dependent, with maximal effect observed in doses of 75 µM and 100 µM of HU-585 (Figure 4B).

Given the observed differential sensitivity of SK-N-SH cells to the various tested compounds, we further examined the in vivo antitumor effect using the nude mice Xn model. Nude immunodeficient mice bearing SK-N-SH Xns expressing mCherry were treated once daily for 21 days with HU-585, ABT-737, a combination of HU-585 and ABT-737 or with a vehicle control. For in vivo studies of senolytic compounds, ABT-737 (75 mg/kg) was used as previously described [23]. It was specifically chosen to be given IP in contrast to its closely related compound ABT-263 used in the in vitro tests that is an orally bioavailable agent. Based on the results of the in vitro studies that demonstrated a better efficacy of HU585 in comparison to its parent compound HU600 at lower doses, the doses selected for HU585 for the in vivo studies (120 mg/kg) were lower than the doses reported for HU600 treatment (200 mg/kg) in animal experiments [21]. Body weight change was assessed as an indicator of side effects and treatment toxicities. No significant weight loss was observed in any treatment group, indicating that this dosing strategy and the dosing intervals used were well tolerated and safe (Data not shown). The average total radiant efficiency signal as measured by IVIS was used to evaluate Xn response to treatment and tumor volume. At the end of treatment, the growth rate and volume of the Xns in the mice that were treated with the combination of HU-585 and ABT-737 were significantly lower in comparison to control or HU585 alone as shown in Figure 4C,D (*p* < 0.05). Moreover, the regression in tumor volume that was observed in the combined treatment group contrasted with the other groups in which progression was demonstrated. Together, the results suggest additive in vitro and in vivo anti-tumorigenic effect of combined treatment of HU-585 with the anti Bcl-2 compounds ABT-263 and ABT-737. 

## 4. Discussions

The endocannabinoid system is currently under intense investigation due to the therapeutic potential of endocannabinoids as treatment options for cancer. Structural modifications of these substances are under investigation and synthesis of novel derivatives with better properties is being explored. Therefore, the aim of this study was to investigate the anti-tumorigenic effect of HU-585, a novel compound obtained by a combination of features of the endocannabinoid anandamide and the drug Minerval (HU-600), expecting to potentiate the antitumorigenic effects of HU-600 against neuroblastoma, an aggressive and resistant pediatric tumor in which identification of new therapeutic strategies are needed.

Minerval, a nontoxic synthetic analog of oleic acid (OA), represents a new class of orally bioavailable lipids used for membrane lipid therapy (MLT). MLT is a new rapidly evolving approach for treating cancer, in which the cellular membranes rather than specific proteins constitute the therapeutic target [22].

In the search for molecules capable of regulating membrane lipid structure, oleic acid was found to be the most active in many types of cancers [24]. However, the therapeutic effect of oleic acid is limited due to its rapid metabolism [25]. In contrast, its synthetic analog, Minerval, is believed to have a more long-lasting pharmacological effect, which favors its therapeutic effect [26]. Minerval has been shown to restore the normal membrane lipid structure and composition in certain tumor cells [27] and by this to inhibit membrane protein-associated aberrant signaling pathways, such as RAS/MAPK and PI3K/AKT pathways [28]. By contrast, Minerval does not significantly alter membrane lipid composition in non-tumor cells, which explains its specificity for cancer cells and the lack of undesired side effects [29]. Moreover, the difference in IC50 values between normal cells (>5000 μM) and cancer cells (30–200 μM) and minimum lethal dose >3000 mg/kg in rats indicate that the therapeutic window for this drug is far below the maximum tolerated dose (or minimum lethal dose), unlike most anticancer drugs currently used. These facts support the specificity of Minerval and its use as a therapy agent to treat cancer. This efficacy and lack of toxicity at therapeutic doses has been acknowledged by the European Medicines Agency (EMA) to designate 2OHOA orphan drug for the treatment of glioma [16].

In a similar way, endocannabinoids, such as AEA are lipid-based derivatives that demonstrate anti-tumorigenic effects mediated by modulation of the ERK and AKT signaling pathways [30,31]. We have previously shown that the ethanolamides of fatty acids have a better anticancer profile than the acids themselves [32]; hence, we synthesized and tested the ethanolamide derivative of Minerval. The novel compound, chemically closely related to Minerval, also resembles the endocannabinoid anandamide (AEA) found in the mammalian body [33]. As Minerval and AEA exert similar protective effects against cancer, we assumed that a molecule that is a derivative of both may have a better anticancer effect while maintaining the high safety profile typical of these two compounds.

To investigate the anti-tumorigenic effects and the mechanisms behind the effects of Minerval (HU-600) and the novel derivate HU-585 on SK-N-SH NBL cell line, cell viability, CFU, and migration in response to treatment were first analyzed followed by apoptosis and senescence studies.

We found that treatment with either HU-600 and HU-585 had an antiproliferative effect as demonstrated by MTT and by CFU assays. HU-600 had only a modest effect on the viability of these cells with a significantly better effect of the derivate HU-585. In addition, as membrane lipid composition has been shown to influence cancer cell migration abilities [34,35,36] we next explored whether HU-600 and its derivative HU-585 can disrupt the migratory ability of NBL cells, a crucial step in the metastatic process and in tumor dissemination. Using migration assay, we found that HU-585 had a better antimigratory effect on NBL cells in comparison to HU-600. Therefore, the conversion of HU-600 to its ethanolamide, leading to HU-585, increases the pharmacological potency of this drug, regarding viability and migration ability of NBL cells.

Impaired apoptosis plays an important role in tumorigenesis and tumor resistance to oncologic treatment [37]. The mechanism of apoptosis is evolutionarily conserved and is executed by a family of proteins called caspases, whose activation is mainly regulated by the anti- apoptotic Bcl-2 family proteins, including Bcl-2, Bcl-XL, Bcl-w, Mcl-,1 and Bfl-1/A1 [38]. HU-600 has been shown to induce apoptosis in several cancer cell lines [39], hence we sought to explore apoptosis as a possible anti-tumorigenic mechanism of its derivative HU-585. We showed that HU-585 induces apoptosis as demonstrated by increased levels of active caspase-3 and a decreased expression of Bcl-2, both markers of apoptosis [40,41,42].

Bcl-2 expression was reported to be strongly increased in most NBL tumors, supporting that Bcl-2 antagonists may have clinical utility for a large subset of patients [43]. Indeed, preclinical studies using Bcl-2-specific inhibitors have demonstrated anti-tumor activity in neuroblastoma tumors with high Bcl-2 levels. Currently, Bcl-2 inhibitors in combination with classical cytostatic drugs are being investigated in clinical trials for the treatment of relapsed or refractory neuroblastoma [44]. 

Many oncogenic stimuli leading to apoptosis can also induce senescence, which is a special state of durable cell cycle arrest. Hence, further to our observation related to the proapoptotic properties of HU-585, we decided to explore whether HU-600 and HU-585 also induce senescence in SK-N-SH NBL cell line. Our results demonstrate that cells treated with HU-585 exhibited senescence in contrast to cells treated with HU-600. Senescence is generally regarded as a tumor suppressive process which evolved alongside apoptosis to suppress tumorigenesis and tumor progression and is considered as an important alternative cell fate to apoptosis [45,46]. Thus, one way to enhance anti-cancer treatment is to use compounds that induce senescence. To this end, senescence-inducing compounds have been developed, including CDK4/6 inhibitors, such as abemaciclib, palbociclib, and ribociclib [47,48]. With respect to neuroblastoma, LEE011, a highly specific CDK4/6 inhibitor caused cell-cycle arrest and cellular senescence in a large subset of neuroblastoma cell line and Xn models [49]. Because this class of drugs has shown promise in treating several cancers in pre-clinical and clinical studies [47,48,49], high-throughput screens have been employed to find additional drug targets that trigger senescence in cancer cells [50]. Based on our present findings, we think that HU-585 can also be considered as a senescence-inducing compound and is a promising candidate for further testing and implementation in current treatment protocols of neuroblastoma patients. 

Paradoxically, senescence has also been proposed to have pro-tumorigenic effects. Accumulating evidence indicates that, following treatment, senescent tumor cells promote tumor relapse, aggressiveness, and metastases via upregulation of antiapoptotic mechanisms [51] and by secretion of cytokines and growth factors that may promote the proliferation of tumor cells [52]. In contrast to apoptosis, senescent cells are stably viable and have the potential to influence neighboring cells through secreted soluble factors, known as the senescence-associated secretory phenotype (SASP), which may have pro-neoplastic properties, including angiogenesis, epithelial—mesenchymal transitions, and differentiation within the local microenvironment [53,54,55,56].

Although senescence induction in cancer cells is a potential therapeutic option to reduce initial tumor growth, it seems to be an imperfect tumor-suppressive treatment. Hence, we assume that chronically persisting senescent cells should be removed by senolytic drugs, which selectively destroy such cells. These drugs can presumably be given in combination with other cancer therapies in order to minimize progression risk and avoid deleterious side effects [57].

It has been shown that overexpression of Bcl-2 counteracts the pro-apoptotic genes during senescence [58]. Senolytic drugs, which target the anti-apoptotic signaling through Bcl-2 family members, (Navitoclax/ABT-263 and ABT-737), were shown to be effective in inducing cell death in senescent cells [59]. As manipulation of the anti-apoptotic Bcl-2 family proteins can influence the choice between senescence and apoptosis, we decided to explore whether treatment with the combination of HU-585 with anti Bcl-2 compounds can have a better anti-tumorigenic effect compared with each drug alone in vitro and in vivo. We have shown that treatment of SK-N-SH NBL cells with a combination of anti Bcl-2 compounds and HU-585 resulted in enhanced anti-proliferative effect in vitro and reduced Xns growth in vivo in comparison to treatment with HU-585 alone.

## 5. Conclusions

In the search for novel therapeutic options in cancer treatment, it has become increasingly clear that in addition to targeting specific proteins, the cellular membrane constitutes an attractive target. In accordance with our findings, we conclude that the endocannabinoid-like substance HU-585, used for MLT, can halt the growth and dissemination of neuroblastoma cancer cells through apoptosis and senescence induction. Although senescence is beneficial for arresting apoptosis-resistant cancer cells, inducing senescence in other situations promotes cancer relapse and secondary tumors. Based on our present findings of enhanced anti-tumorigenic effects of combination therapy by HU-585 with senolytic drugs, we propose that this approach might provide a novel complementary and less toxic therapeutic strategy for the treatment of refractory neuroblastomas. Further studies are needed to validate the efficacy of this novel approach and to explore its advantage over current established treatments.

## Data Availability

Data is contained within the article.
